# Aging alters antiviral signaling pathways resulting in functional impairment in innate immunity in response to pattern recognition receptor agonists

**DOI:** 10.1007/s11357-022-00612-5

**Published:** 2022-07-18

**Authors:** Jennifer Connors, Bhavani Taramangalam, Gina Cusimano, Matthew R. Bell, Stephanie M. Matt, Kaitlyn Runner, Peter J. Gaskill, Victor DeFilippis, Janko Nikolich-Žugich, Michele A. Kutzler, Elias K. Haddad

**Affiliations:** 1 Department of Medicine, Division of Infectious Diseases & HIV Medicine, Drexel University College of Medicine, Philadelphia, PA USA; 2 Department of Microbiology and Immunology, Drexel University College of Medicine, Philadelphia, PA USA; 3 Department of Pharmacology and Physiology, Drexel University College of Medicine, Philadelphia, PA USA; 4 Vaccine and Gene Therapy Institute, Oregon Health and Science University, Portland, OR USA; 5 Department of Immunobiology, University of Arizona College of Medicine-Tucson, Tucson, AZ USA; 6 Arizona Center On Aging, University of Arizona College of Medicine-Tucson, Tucson, AZ USA

**Keywords:** Aging, Vaccines, Antiviral, Interferons, Dendritic cells, Monocytes, Phagocytosis

## Abstract

The progressive impairment of immunity to pathogens and vaccines with aging is a significant public health problem as the world population shifts to an increased percentage of older adults (> 65). We have previously demonstrated that cells obtained from older volunteers have delayed and defective induction of type I interferons and T cell and B cell helper cytokines in response to TLR ligands when compared to those from adult subjects. However, the underlying intracellular mechanisms are not well described. Herein, we studied two critical pathways important in the production of type I interferon (IFN), the interferon response factor 7 (pIRF7), and TANK-binding kinase (pTBK-1). We show a decrease in pIRF7 and pTBK-1 in cross-priming dendritic cells (cDC1s), CD4^+^ T cell priming DCs (cDC2s), and CD14^dim^CD16^+^ vascular patrolling monocytes from older adults (*n* = 11) following stimulation with pathway-specific agonists in comparison with young individuals (*n* = 11). The decrease in these key antiviral pathway proteins correlates with decreased phagocytosis, suggesting impaired function in Overall, our findings describe molecular mechanisms which explain the innate functional impairment in older adults and thus could inform us of novel approaches to restore these defects.

**Graphical abstract:**

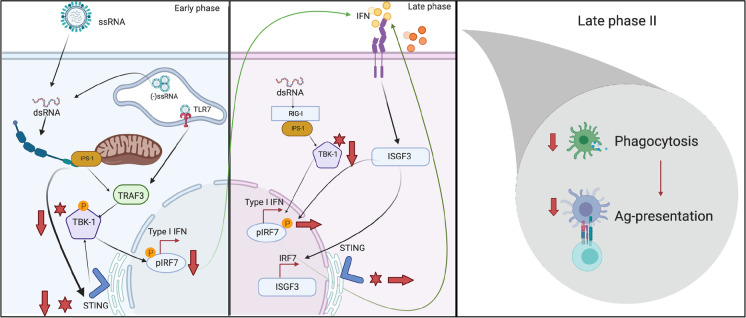

**Supplementary Information:**

The online version contains supplementary material available at 10.1007/s11357-022-00612-5.

## Introduction

The progressive decline in the function of the immune system with increasing age is a condition known as *immunosenescence*. Unsurprisingly, this decline leads to decreased protection in the aging population against infectious pathogens [1]. This deterioration is evidenced in the mortality rates of this age group with almost 85% of pneumonia and influenza-associated deaths in the USA, occurring in individuals over the age of 65 [2]. The onset of the COVID-19 pandemic further underscored this vulnerability as individuals aged 65–74 were more than 95 times more likely to die from the virus when compared to the 18–29-year-old population [3]. With the global population over the age of 65 expected to double by 2050 [4], it is imperative to gain a better understanding of the underlying mechanisms of immunosenescence. Paradoxically, although chronically elevated low levels of inflammation in the absence of overt infection, also called *inflammaging*, are characteristic of older adults people [5], peripheral blood mononuclear cells (PBMCs) from older adults demonstrate a decrease in the production of inflammatory and antiviral cytokines upon Toll-like receptor (TLR)7/8 and retinoic acid-inducible gene I (RIG-I) stimulation when compared to their younger counterparts [6]. Our group has previously shown this effect in monocyte subsets sorted from PBMCs of our aging cohort in response to TLR7/8 and RIG-I stimulation, resulting in the decreased induction of protein and gene expression of interferon (IFN)-γ and interleukin-1β (IL-1β). RIG-I stimulation also resulted in decreased production of IFN-α by monocytes from older individuals [7].

Monocytes are a critical early line of defense against invaders, rapidly trafficking to the sites of infection and differentiating into professional antigen-presenting dendritic cells (DCs) and macrophages. Aside from being a major source of antiviral and inflammatory cytokines and chemokines [8–10], these cells are important in bridging innate and adaptive immunity, acting as antigen presenters for the priming and activation of cytotoxic and helper T cells [11]. DCs are essential for the germinal center (GC) reaction in the lymph nodes, where activated follicular helper T (Tfh) cells recognize and bind cognate B cells and drive somatic hypermutation and affinity maturation of B cell antibodies [12].

Most studies investigate monocytes or DCs as a whole lineage, but both cell types can be split into functionally distinct subsets that each plays important roles in antiviral defense and other functional roles such as phagocytosis and antigen presentation. Conventional type 1 DCs (cDC1) are the primary subset that cross-presents antigen to CD8^+^ T cells and dominantly produce IL-12 [13–16]. In contrast, conventional type 2 DCs (cDC2) have been associated with CD4^+^ T_H_ cell responses including GC T_FH_ responses [16–18]. Plasmacytoid DCs (pDCs) are from a different lineage than cDCs and resemble plasma cells. These cells secrete large quantities of type I IFN in response to viral infection [19]. Monocytes also represent a heterogeneous population with three distinct subsets classified by the expression of CD14 and CD16. Classical monocytes (CD14^+^CD16^−^) are the most abundant and produce high levels of reactive oxygen species in response to pathogens [20]. The other minor populations are divided into two subsets: intermediate (CD14^+^CD16^+^) and non-classical monocytes (CD14^dim^CD16^+^). CD14^+^CD16^+^ monocytes are producers of high levels of reactive nitrogen species (RNS), IL-1B, and TNF-α while CD14^dim^CD16^+^ monocytes patrol the vascular endothelium in response to virus and produce TNF-α and IL-1β [20, 21].

DCs and monocytes help to shape the innate immune response via the activation of pattern recognition receptors (PRRs) such as TLR7/8 and RIG-I. Upon infecting a cell, viruses like SARS-CoV-2 may activate PRRs including TLR7/8, by nature of their single-stranded RNA, or RIG-I, through its double-stranded RNA replication intermediates [22–26]. TLR7/8 and RIG-I activation initiates a series of signaling events that are mediated by TANK-binding kinase 1 (TBK-1), which leads to the phosphorylation, dimerization, and translocation of the transcription factor, IRF7 [27, 28]. Identifying deficits in any of the pathways responsible for IFN production may lead to elucidating potential avenues for therapeutic targets.

Using PBMCs from young (24–36-year-old) and older healthy adult (67–83-year-old) donors, we sought to determine whether certain subsets of monocytes and DCs have impairments in antiviral signaling and how those deficits affect the functions of the cells. We determined that decreased phosphorylation of IRF7 and TBK-1 in CD14^dim^CD16^+^ monocytes, cDC1, and cDC2 and a subsequent decrease in STING activation in older adults was associated with the impaired primary IFN induction during the early phase of simulated viral infection. We further observed that the defective induction of IFN led to a decrease in phagocytosis. These results contribute to the knowledge of the impact of aging on innate antiviral function and benefit the field by delineating precise age-related defects in specific subsets of monocytes and DCs, individually.

## Experimental methods

### Human samples

Blood samples were obtained from healthy donors at Martin Memorial Health Systems (FL). Consenting adults were screened using a questionnaire determining their demographic information, medication usage, and comorbidities. Participants were excluded with any acquired immunodeficiency or immunomodulating medications (such as steroids, chemotherapy, or history of autoimmune disease), pregnancy, history of cancer and history of cirrhosis or renal failure, or antibiotic use within 2 weeks of recruitment. Blood samples were taken from individuals aged 18–80 and were made into single-cell PBMC suspensions and frozen in bovine serum albumin (Sigma) plus 10% DMSO (VWR) for cryopreservation in liquid nitrogen. The institutional review boards at the relevant institutions approved all procedures, and all participants provided signed informed consent.

### In vitro stimulation of monocyte and DC subsets

PBMCs from healthy young and older donors were plated at a volume of 1.0 × 10^6^ cells per well in a round 96-well plate in a volume of 100 µl of complete RPMI medium (RPMI 1640 with l-glutamine [Corning Cellgro, Manassas, VA] supplemented with 10% FBS and 13 [50 U] penicillin–streptomycin [Invitrogen, Carlsbad, CA]). For experiments involving LPS/IFN-α or IFN-γ stimulation, PBMCs were stimulated for 15 min, 45 min, or 24 h with 1 µg/ml LPS with 80 ng/ml (InvivoGen, Cat# TLRL-eblps) and IFN-γ (InvivoGen, Cat# 300-134P). For experiments involving RIG-I agonist, PBMCs were stimulated for 24 h with 500 ng/ml of a RIG-I ligand, 3p-hpRNA/LyoVec (InvivoGen, Cat# tlr-hprnalv). A LyoVec-only control was used for this stimulation. For experiments involving a cyclic GMP-AMP synthase (cGAS)-STING agonist, PBMCs were stimulated for 24 h with 75 µM of G10 STING agonist provided to us by Dr. Vincent DeFillipis at the Vaccine and Gene Therapy Institute at Oregon Health and Science University and DMSO control was used. Optimal concentrations of different TLR agonists were selected based on the median production of IL-6 and IFN-α and an 85% or more survival rate of monocytes. All PRR ligands were purchased commercially (InvivoGen, San Diego, CA) except for the G10 STING agonist. Where indicated, supernatants were collected after stimulation and frozen at − 80 °C.

### Phosflow cytometry analysis of monocyte and DC subsets

The stimulated PBMCs from adult or older adult donors were prepared and incubated with fluorochrome-conjugated antibodies for flow cytometry. Briefly, after stimulation, cells were washed twice with fluorescence-activated cell sorting (FACS) buffer (PBS containing 2% FBS); surface stained using antibodies for 30 min in 100 µl of FACS buffer; permeabilized using 300 µl of cold BD Phosflow buffer III (BD Biosciences) according to the manufacturer’s instructions; intracellular phosphoprotein stained using antibodies against intracellular-phosphorylated IRF7 (pS477/pS479, BD Biosciences), pTBK-1 (BD Biosciences, or STING (BD Biosciences in 50 µl FACS buffer for 1 h); and then fixed using 2% PFA for 15 min at 37 °C. The following fluorochrome-conjugated antihuman antibodies were used: CD3 (clone: HIT3α, Cat: 300324), CD56 (clone: 5.1H11, Cat: 362504), CD19 (clone: HIB19, Cat: 302216), CD20 (clone: 2H7, Cat: 302311), CD11c (clone: BU15, Cat: 337110), CD16 (clone: 3G8, Cat: 302026), CD14 (clone: M5E2, Cat: 301822), HLA-DR (clone: L243, Cat: 307626), CD1c (clone: L161, Cat: 331519), and CD303 (clone: 201A, Cat: 354212), which were all from BioLegend. pIRF7 (clone: K47-671, Cat: 558630), pTBK-1 (clone: J133-587, Cat: 558604), and STING (clone: T3-680, Cat: 564836) were all from BD Biosciences. CD141 (clone: AD5-14H12, Cat: 130–113-317) was from Miltenyi Biotech. LIVE/DEAD Fixable Dead Cell Stain (Life Technologies, Cat: L34957) was used to gate on live cells. Samples were acquired on a BD™ LSRFortessa (BD Biosciences), and analysis was conducted using FlowJo software (version 10). Cells were phenotyped as follows: cDC2 were lineage^−^(CD19^−^CD3^−^CD56^−^CD20^−^)HLA-DR^+^CD11c^+^CD1c^+^CD141^−^CD303^−^, cDC1 were lineage^−^HLA-DR^+^CD11c^−^CD1c^−^CD141^+^CD303^−^, pDCs were lineage^−^HLA-DR^+^CD11c^−^CD1c^−^CD141^−^CD303^+^, classical monocytes were lineage^−^HLA-DR^+^CD14^+^CD16^−^, intermediate monocytes were lineage^−^HLA-DR^+^CD14^+^CD16^+^, and non-classical monocytes were lineage^−^HLA-DR^+^CD14^dim^CD16^+^.

### In vitro phagocytosis assay

PBMCs from healthy young and older donors were plated at a volume of 1.0 × 10^6^ cells per well in a round 96-well plate in a volume of 100 µl of complete RPMI medium at 37 °C and at 4 °C as a negative control. PBMCs were then stimulated as stated previously except all stimulations occurred for 24 h. After the 24-h stimulation period, cells were washed twice with RPMI to remove the agonist and incubated with 0.04-µm fluorescent microspheres (Invitrogen, Cat: F8794) in RPMI for 3 h. The cells were then washed twice with FACS buffer to remove any beads from the outside of the cell and prepared for flow cytometry.

### Phagocytic flow cytometry analysis of monocyte and DC subsets

The PBMCs from adult or older adult donors were prepared and incubated with fluorochrome-conjugated antibodies for flow cytometry. Briefly, after stimulation, cells were washed twice with FACS buffer, surface stained using antibodies for 30 min in 100 µl of FACS buffer, and then fixed using 2% PFA for 15 min at 37 °C. Cells were phenotyped as stated previously.

### Cytokine and chemokine analysis

Supernatants collected from PBMCs during stimulation were analyzed for chemokine/cytokine levels using Life Technologies magnetic bead assays (Invitrogen). Using the human ProcartaPlex™ Panel (Invitrogen™), the following human chemokine–premixed panels was used: IL-1β, IL-6, TNF-α, IFN-α, and IFN-γ. The manufacturer’s protocol was followed. Data were acquired on a Luminex™ FLEXMAP 3D™ System (using bead regions defined in the Invitrogen protocol) and analyzed with the Belysa Curve Fitting Software (Sigma-Aldrich).

### Statistics

All flow cytometry, Luminex, and confocal data were analyzed using GraphPad Prism v9. Where appropriate, stimulations were subtracted from their background controls; i.e., LPS/IFN-γ-stimulated cells were subtracted from unstimulated, the RIG-I agonist was subtracted from LyoVec-only control, and the G10 STING agonist was subtracted from a DMSO control. The unpaired, non-parametric Mann–Whitney *U* test was used when comparing two groups. The paired multiple *t* test and non-parametric one-way ANOVA (Friedman) test were used when comparing more than two groups to each other (*p < 0.05, **p < 0.01, ***p < 0.001, ****p < 0.0001).

## Results

### PBMCs from older individuals display impairment in type I IFN and helper cytokine production

**Fig. 1:**
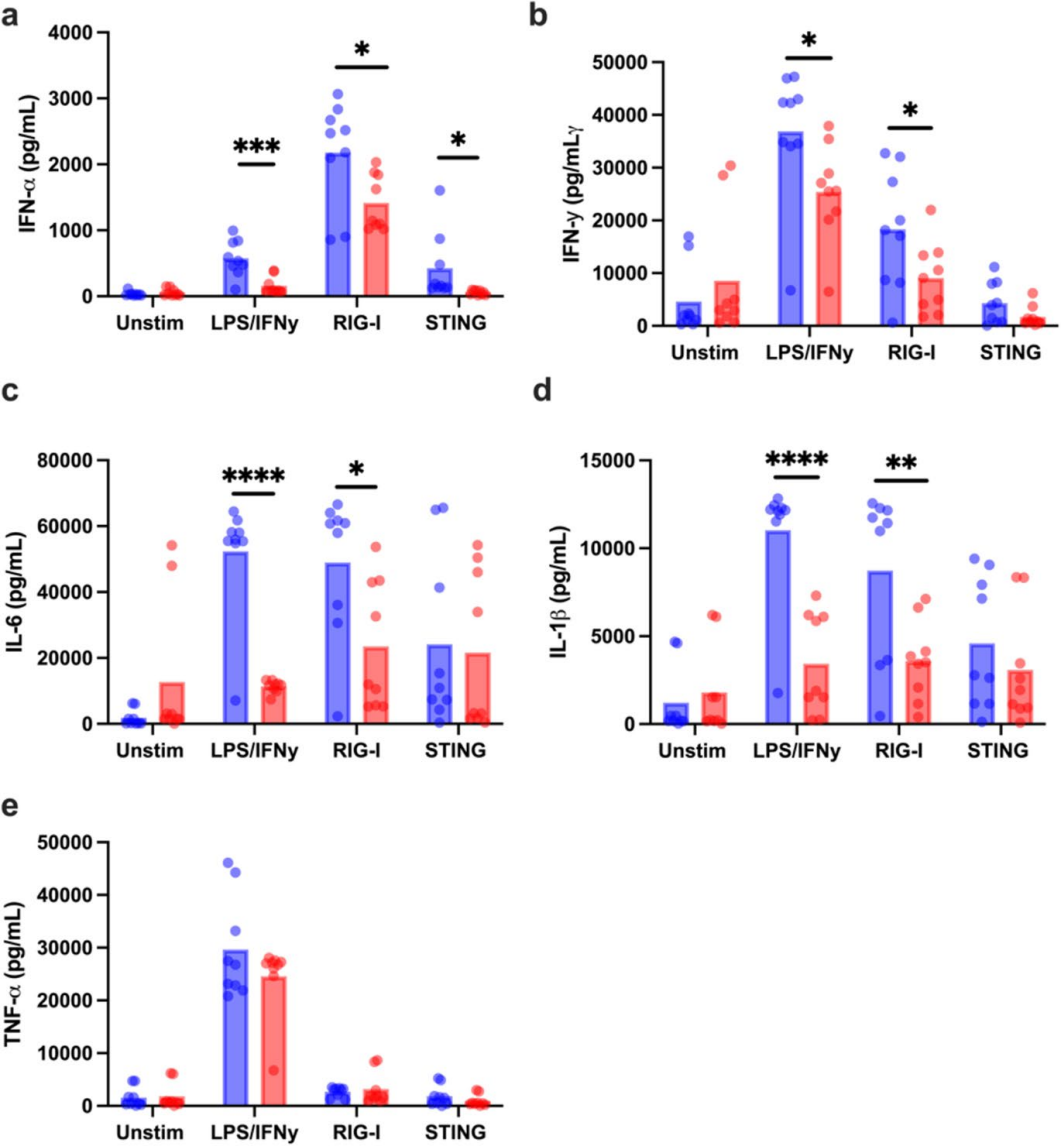
Age negatively affects type I IFN and cytokine production. **a**–**e** Human PBMCs from the blood of younger (age, 24–36 years; *n* = 9) and older (age, 67–83 years; *n* = 9), healthy donors were either stimulated with LPS/IFN-γ, G10, a cGAS-STING-specific agonist or transfected with a retinoic acid–inducible gene I (RIG-I)–specific 3p-hpRNA ligand, or left unstimulated. The unstimulated control for LPS/IFN-γ is complete RPMI, the control for RIG-I is LyoVec only, and the control for G10 is DMSO. The supernatant was collected from PBMCs of all donors 24 h after stimulation and analyzed by Luminex bead-based assay to measure IFN-α (**a**), IFN-γ (**b**), IL-6 (**c**), IL-1β (**d**), or TNF-α (**e**) secretion. All concentrations were measured using the human ProcartaPlex 5 Plex from Invitrogen. Data are means ± SEM. *p < 0.05, **p < 0.01, ***p < 0.001, ****p<0.0001  unpaired, non-parametric Mann–Whitney *U* test

**Fig. 2:**
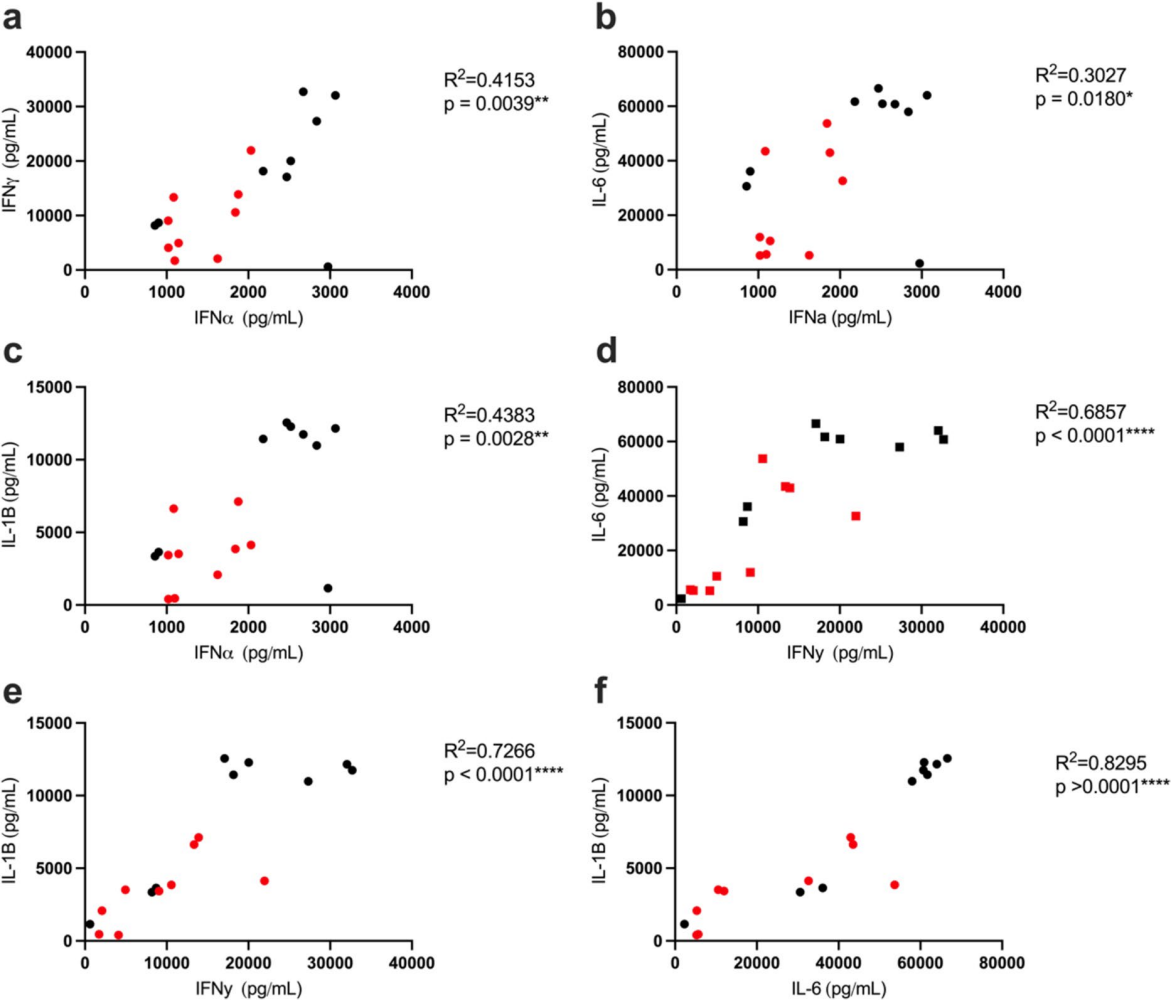
Type I IFN and cytokine production is positively correlated. **a**–**f** The supernatant was collected from PBMCs of all donors (9 old and 9 young) 24 h after stimulation and analyzed by Luminex assay to measure IFN-α, IFN-γ, IL-1β, TNF-α, or IL-6 secretion as described in Fig. 1. Each point represents one donor (red for old and blue for young). Pearson’s correlation test was used for all correlation graphs. *n* = 9/group. *p < 0.05, **p < 0.01, ****p < 0.0001

Healthy individuals enrolled into the study were accrued into two groups: adults and older subjects (*n* = 11 per group) with an equal sex distribution. Individuals with comorbid conditions like cancer within the last 5 years, or other immunocompromising conditions, and steroid use were excluded. Inclusion criteria included controlled hypertension, occasional aching joints from arthritis and not taking daily non-steroidal anti-inflammatory drugs or acetaminophen, and controlled diabetes. The average age for adults was 30 years (range 24–36 years), whereas for older adults, it was 73 years (range 67–83 years) (Supplemental Table 1). We examined the induction of the IFN-α, IFN-γ, IL-1β, TNF-α, and IL-6 in PBMCs from the two groups after 24-h stimulation with LPS/IFN-γ, RIG-I agonist (3p-hpRNA), or cGAS-STING agonist by multiplex bead assay. LPS/IFN-γ and RIG-I agonists are commercially available whereas the cGAS-STING agonist herein termed G10 was developed and provided to us by a group at the Vaccine and Gene Therapy Institute at Oregon Health and Science University [29]. G10 has been found to trigger IRF3/IFN and STING activation in human fibroblasts in an indirect manner, as G10 does not bind to STING [30]. Supernatants from unstimulated (baseline) PBMCs from older donors showed some increase in IL-6, IL-1β, and IFN-γ compared to those from adults but did not reach significance (Fig. 1). However, the antiviral cytokine IFN-α (Fig. 1a) and the pro-inflammatory cytokines IFN-γ, IL-6, and IL-1β (Fig. 1b–d) were significantly secreted at lower levels in PBMCs (*n* = 11 biologically independent donors per group) from older participants compared to adults after stimulation. Specifically, PBMCs from adult and older participants were able to produce IFN-α in response to LPS/IFN-γ, RIG-I, and STING agonists; however, the production of this cytokine was reduced in old individuals in response to all these stimulations (*p* value = 0.0008 for LPS/IFN-γ, *p* value = 0.0141 for RIG-I agonists, and *p* value = 0.0449 for STING agonist) (Fig. 1a). Similarly, IFN-γ was secreted from both groups; however, older participants produced lower levels in response to LPS/IFN-γ (0.0403), RIG-I agonist (0.0482), and STING agonist (0.0992) (Fig. 1b). Additionally, IL-6 and IL-1β production was also detected in both groups; however, at significantly reduced levels in older subjects (*p* < 0.0001 [IL-6] and *p* = 0.0001 [IL-1β] for LPS/IFN-γ stimulation and *p* = 0.0185 [IL-6] and *p* = 0.0076 [IL-1β] for RIG-I stimulation) (Fig. 1c, d). We did not observe age-related differences in IL-6 and IL-1β production in response to STING agonist or in TNF-α production in response to the 3 different stimulations (Fig. 1e). A correlational analysis was also performed to determine whether individuals who produced high levels of a particular cytokine, i.e., IFN-γ in response to RIG-I agonist, similarly produced high levels of any other cytokine such as IFN-α. As expected, most cytokine levels strongly correlated with one another, and this result was independent of age (Fig. 2a–f). Overall, these results suggested an impairment in the ability of PBMCs from older subjects to respond to a variety of innate immune stimulus.

### Aging impairs the phosphorylation of IRF7 in response to TLR7/8 and RIG-I ligation

**Fig. 3:**
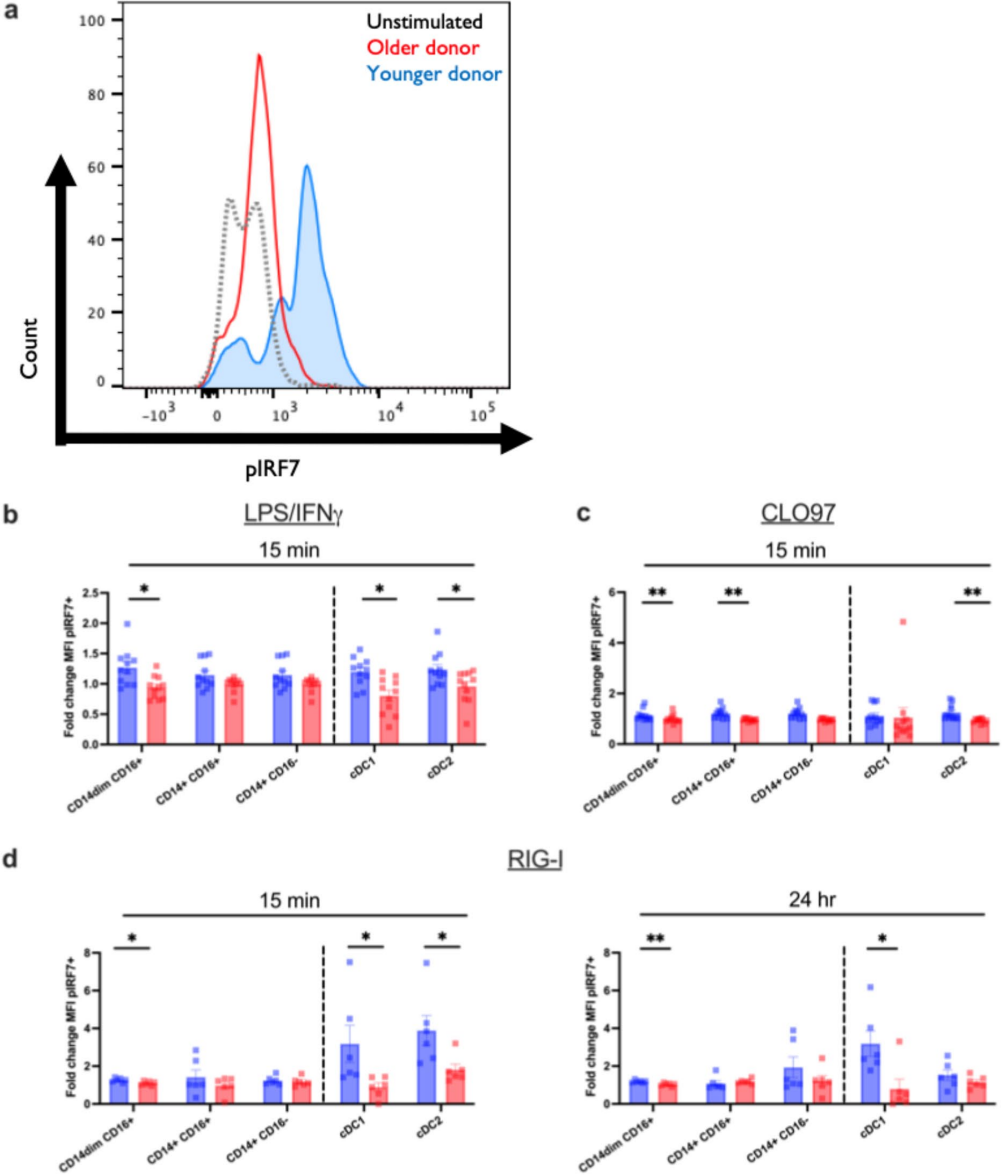
Monocyte and DC subsets from older individuals are impaired in interferon response factor 7 (IRF7) phosphorylation. Human PBMCs from the blood of younger (age, 24–36 years; *n* = 11) and older (age, 67–83 years; *n* = 11) healthy donors were either stimulated with LPS/IFN-γ or CLO97, a TLR7/8-specific agonist, or transfected with a RIG-I-specific 3p-hpRNA ligand. For unstimulated and control conditions, refer to Fig. 1. **a** Representative flow plot for data presented in **b**–**d**. Using these plots, the fold change of pIRF7 MFI was calculated using the unstimulated as the control. **b**–**d** PBMCs were either stimulated with LPS/IFN-γ, CLO97, or RIG-I agonist or left unstimulated for 15 min or 24 h. Cells were permeabilized, fixed, and stained for phosphorylated IRF7 transcription factor. After gating on monocyte and DC subsets, the geometric mean intensity (MFI) was measured using Phosflow cytometry. Data are means. *p < 0.05, **p < 0.01, ***p < 0.001, unpaired, non-parametric Mann–Whitney *U* test

**Fig. 4:**
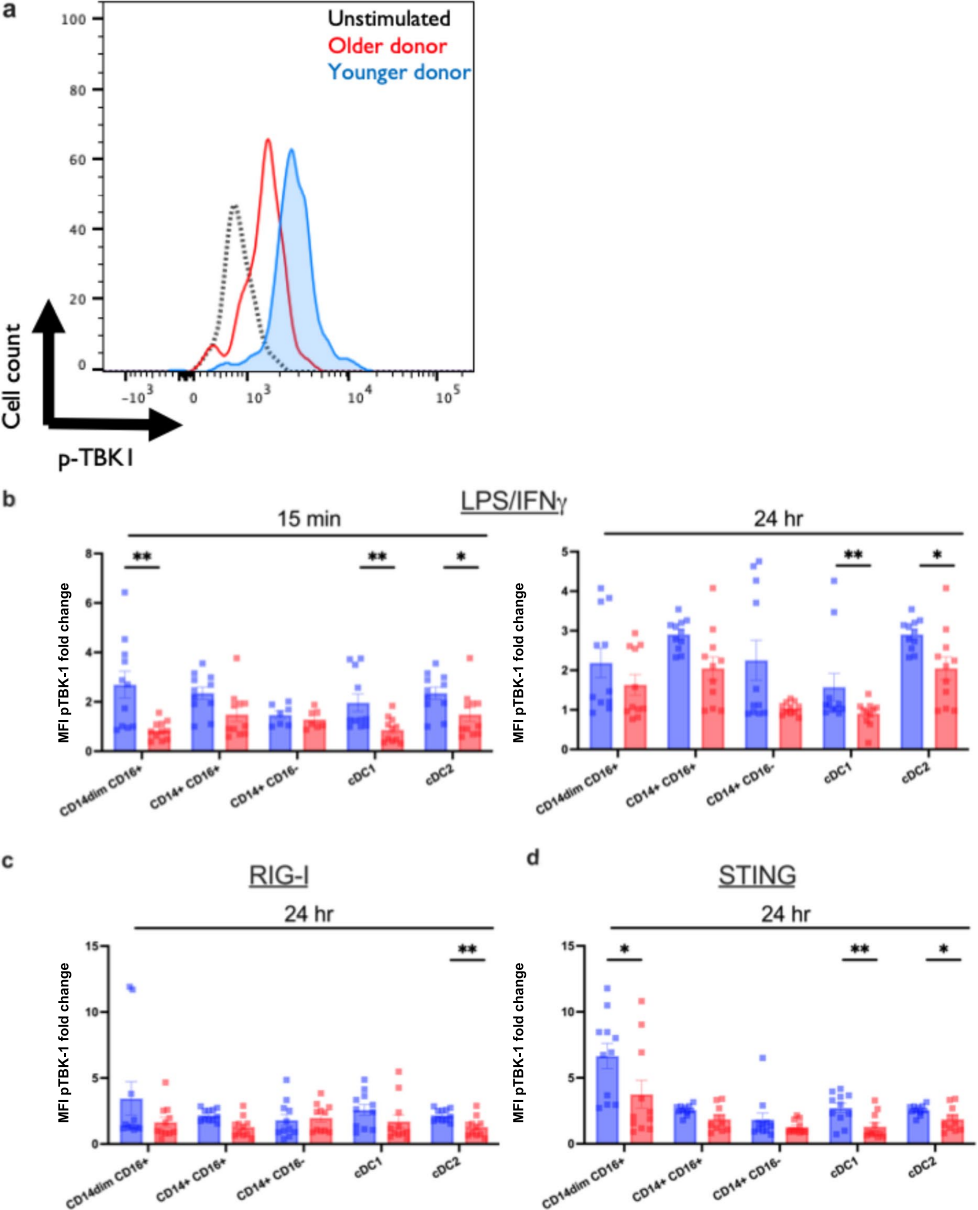
Monocyte and DC subsets from older individuals are impaired in TANK-binding kinase (TBK) phosphorylation. Human PBMCs from the blood of younger (age, 24–36 years; *n* = 11) and older (age, 67–83 years; *n* = 11) healthy donors were either stimulated with LPS/IFN-γ, G10, a cGAS-STING-specific agonist, or transfected with a RIG-I-specific 3p-hpRNA ligand. **a** Representative flow plot for data presented in **b**–**d**. Using these plots, the fold change of pTBK-1 MFI was calculated using the unstimulated as the control. **b**–**d** PBMCs were either stimulated with LPS/IFN-γ, G10, or the RIG-I agonist or left unstimulated for 15 min and 24 h. Cells were permeabilized, fixed, and stained for phosphorylated TBK-1. After gating on DC and monocyte subsets, the MFI was measured using Phosflow cytometry. Data are means. *p < 0.05, **p < 0.01, non-parametric Mann–Whitney *U* test

To analyze the specific defects in viral innate signaling that arise from age, we measured the phosphorylation and activation status of several key molecules in each pathway in response to pathway-specific agonist in peripheral blood monocyte and DC subsets as a function of age. Type I IFN production in response to a single-stranded RNA virus like influenza or SARS-CoV-2 can originate from multiple pathways including the TLR7/8 and RIG-I signaling pathways [31]. We would like to note that the impaired production in older PBMCs was not due to differences in TLR expression on DC and monocyte subsets (Supplemental Fig. 2) or due to altered cell numbers [6, 7, 32]. In fact, examining the same study cohort, we have previously reported that the frequencies and the absolute numbers of the monocyte and dendritic cell subsets are not changed between adult and older donors [7]. Thus, we reasoned that the defect in type I IFN production could be due to impaired intracellular signaling. We first investigated at which point in the IFN induction pathway the monocytes and DCs failed to signal. Unstimulated cells showed similar IRF7 phosphorylation regardless of age (Supplemental Fig. 4). Figure 3a depicts the raw mean fluorescence intensity (MFI) of a representative subject showing levels of pIRF7 in unstimulated (black, dotted) PBMCs from a young donor and an apparent increase in MFI only in adult participants (blue) after the 24-h stimulation with our RIG-I agonist compared with older adult donors (red). After stimulation with LPS/IFN-γ (TLR4), CLO97 (TLR7/8), or 3p-hpRNA/LyoVec (RIG-I) at 15 min (Fig. 3b–d) and a later time point (24 h for RIG-I, Fig. 4d), the subsequent phosphorylation of IRF7 was found to be significantly lower in CD14^dim^CD16^+^ monocytes, CD14^+^CD16^+^ monocytes, cDC1, and cDC2 subsets when compared with healthy adult controls (Fig. 3a–d). Specifically, we observed a significant pIRF7 increase in cDC1 from adults in response to LPS/IFN-γ (*p* < 0.0026) and RIG-I agonist (*p* = 0.0087) after 15 min and the pIRF7 levels persisted in adults after the 24-h RIG-I stimulation (*p* = 0.0260). In cDC2 however, we observed a significant increase in pIRF7 in adults in response to LPS/IFN-γ (*p* < 0.0238), CL097 (*p* < 0.0029), and RIG-I agonist (*p* = 0.0152) at 15 min. IRF7 phosphorylation in monocyte subsets is depicted in Fig. 3b–d. We observed significant pIRF7 in adults in response to LPS/IFN-γ (*p* = 0.0618), CL097 (*p* = 0.0009), and RIG-I agonist (*p* = 0.0087) in CD14^dim^CD16^+^ at 15 min that is maintained in RIG-I-stimulated cells after 24 h. We also observed significant pIRF7 phosphorylation in CD14^+^CD16^+^ in adults compared to older participants (*p* = 0.0391) following stimulation of CL097. At 45 min, there was no difference between pIRF7 levels after stimulation with LPS/IFN-γ and CLO97 (data not shown). These data suggested that IRF7 phosphorylation could be impaired in different monocyte and dendritic cell subsets in older individuals, and this could affect the response production of antiviral and helper cytokines.

### Aging impairs the TBK-1 phosphorylation in response to TLR7/8 and RIG-I ligation

Because of the level of cross-talk associated with the antiviral pathways, we examined TBK-1, a key kinase in the RIG-I and cGAS-STING pathway that directly phosphorylates IRF3 and IRF7 after sensing either a dsRNA intermediate or dsDNA [33]. Similar to what we observed for the phosphorylation of IRF7, unstimulated cells showed similar TBK-1 phosphorylation except in cDC2, where levels were increased in older donors compared to adults (*p* = 0.0400) (Supplemental Fig. 4b). Figure 4a depicts a raw MFI flow plot showing levels of pTBK-1 in unstimulated (black dotted line) PBMCs from an adult donor and an increase in MFI in adult (blue) compared to an older donor (red) after the 24-h stimulation with G10, our STING agonist. After stimulation with LPS/IFN-γ (TLR4) (Fig. 4b) and 3p-hpRNA/LyoVec (RIG-I) or G10 (cGAS-STING)-specific agonists at 24 h (Fig. 4b–d), the subsequent phosphorylation of TBK-1 was impaired in cDC1, and cDC2 dendritic cells and CD14^dim^CD16^+^ monocytes in older participants when compared with adult controls (Fig. 4b–d), suggesting a potential for impaired cross-presentation and activation of CD8^+^ T cells or polarization of CD4^+^ T cells through the secretion of IL-12 during a viral infection [11, 34]. Specifically, we observed a significant pTBK-1 increase in cDC1 from young subjects in response to LPS/IFN-γ (*p* = 0.0096) after 15 min. That difference persisted after 24 h of stimulation with LPS/IFN-γ (*p* = 0.0796) and was significantly increased in response to the G10 STING agonist (*p* = 0.0013). We did not see an increase after stimulation with the RIG-I agonist for 24 h (*p* = 0.1964). In cDC2, we observed that there was a significant increase in pTBK-1 of younger donors across all stimulations including LPS/IFN-γ at 15 min (*p* = 0.0277) and LPS/IFN-γ (*p* = 0.0128), RIG-I agonist (*p* = 0.0152), and STING agonist (*p* = 0.0301) at 24 h post-stimulation. TBK-1 phosphorylation in monocyte subsets is shown in Fig. 4b–d. We observed significant increases in the phosphorylation of TBK-1 in response to LPS/IFN-γ at 15 min (*p* = 0.0034) which did not persist after 24 h post-stimulation (*p* = 0.1513) and in response to the STING agonist after the 24-h stimulation (*p* = 0.0129) in CD14^dim^CD16^+^ monocytes when compared with adult counterparts. Significant decreases in the phosphorylation of TBK-1 after RIG-I stimulation with aging suggest that the impaired phosphorylation of IRF7 in the RIG-I pathway is a consequence of impaired upstream TBK-1 activation.

### Induction of the cGAS-STING pathway is impaired with age

The most pronounced output of IFN in response to cytosolic DNA accumulation or DNA viral infection is due to the cGAS-STING pathway [35]. In the cGAS-STING pathway, DNA-sensing receptor cGAS is activated by cytosolic DNA that leads to the endogenous generation of the second messenger cyclic GMP-AMP which then binds to STING and activating TBK-1, resulting in the production of type I IFN [36].

**Fig. 5:**
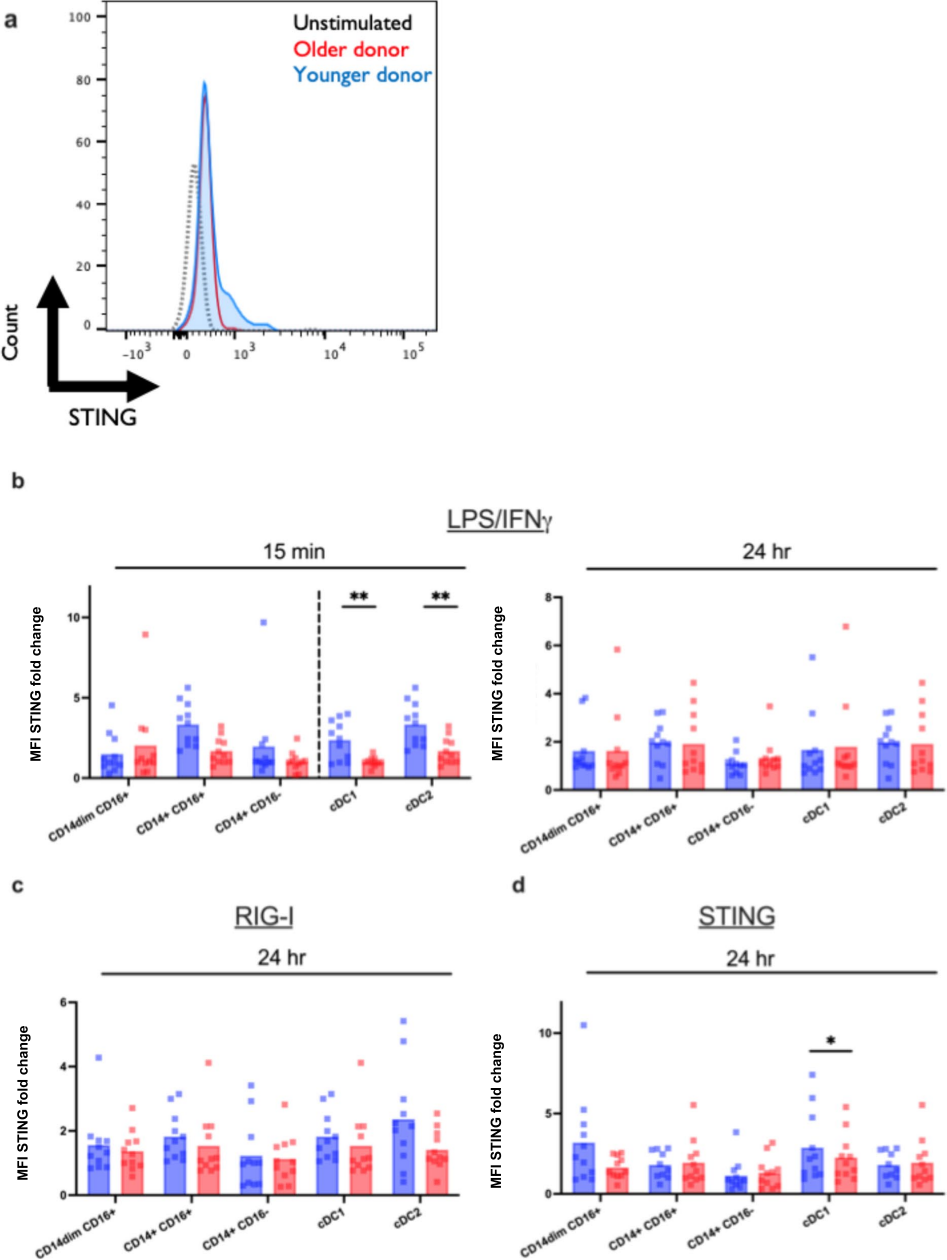
Monocyte and DC subsets from older individuals are impaired in cGAS-STING activation. Human PBMCs from the blood of younger (age, 24–36 years; *n* = 11) and older (age, 67–83 years; *n* = 11) healthy donors were either stimulated with LPS/IFN-γ, G10, a cGAS-STING-specific agonist, or transfected with a RIG-I-specific 3p-hpRNA ligand. **a** Representative flow plot for data presented in **b**–**d**. Using these plots, the fold change of STING MFI was calculated using the unstimulated as the control. **b**–**d** PBMCs were either stimulated with LPS/IFN-γ, G10, or the RIG-I agonist or left unstimulated for 15 min and 24 h. Cells were permeabilized, fixed, and stained for STING. After gating on DC and monocyte subsets, the median intensity (MFI) was measured using flow cytometry. Data are means. *p < 0.05, **p < 0.01, *p* < 0.001, non-parametric Mann–Whitney *U* test

Here, we examined STING activation in response to G10. To determine this, we performed phosphorylation flow cytometry staining of LPS/IFN-γ, RIG-I, and cGAS-STING activation. Figure 5a shows an example of raw MFI data showing unstimulated levels (black, dotted), and increased activation of STING in an adult (blue) compared to an older participant (red) in response to the G10 agonist after 24 h. After stimulation with LPS/IFN-γ (TLR4) (Fig. 5b), the subsequent activation of STING was impaired in cDC1 (*p* = 0.0018) and cDC2 (*p* = 0.0024) at 15 min post-stimulation. After 24 h post-stimulation with G10, the defect in STING expression did not persist in cDC1 or cDC2 after stimulation with either LPS/IFN-γ, RIG-I, or G10 STING agonist. This initial deficit in the STING pathway in cDC1 and cDC2 has implications in antiviral activity against emerging alphaviruses like Chikungunya virus, Venezuelan equine encephalitis virus, and Sindbis virus, for which this pathway has specifically been shown to protect against with activation [37].

### Phagocytosis in monocytes and DCs from older donors is impaired in response to pathway-specific stimulus

**Fig. 6:**
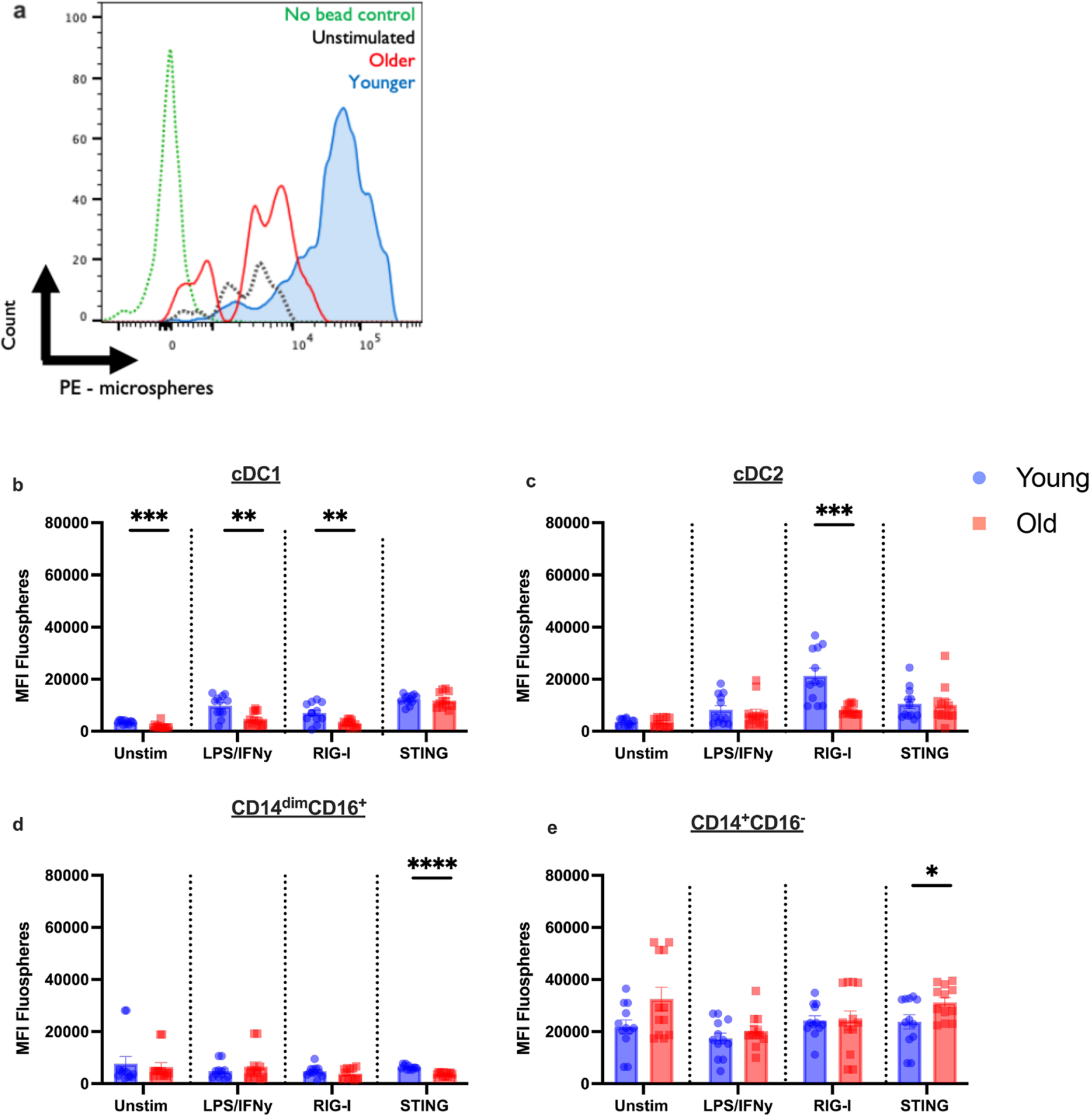
Phagocytosis in response to pathway-specific stimulus is impaired in multiple innate immune subsets from older adults. **a**–**e** Phagocytosis is less efficient in cDC1 (**b**), cDC2 (**c**), CD14^dim^CD16^+^ monocytes (**d**), and CD14^+^CD16.^−^ monocytes (**e**). Phagocytosis was measured using fluorescent beads and multiparametric flow cytometry. PBMCs were incubated overnight with a stimulus and with beads for a further 3-h period. MFI of fluorescent beads of indicated subsets from young (*n* = 11) and old (*n* = 11) patients is indicated. Data is from two independent experiments and are shown (mean ± SEM). *p < 0.05, **p < 0.01, ***p < 0.001, ****p < 0.0001 The *p* values were determined using the non-parametric Mann–Whitney *U* test

Defects in primary TLR7/8, RIG-I, and cGAS-STING signaling caused by the decrease in phosphorylation of transcription factors and molecules like IRF7, TBK-1, and STING could lead to defects in functional roles like phagocytosis, dendritic cell maturation, and antigen presentation. We performed a phagocytosis assay by stimulating PBMCs for 24 h with pathway-specific agonists and incubating with fluorescent beads (0.04 µm) for a further 3-h period to examine the efficiency of being taken up by cells. Figure 6a depicts a representation of our phagocytosis assay as a measure of MFI change after 24 h of stimulation with the G10 agonist. The green dotted line represents the no bead control whereas the black dotted represents our unstimulated condition, and the red and blue histograms represent an older and an adult donor, respectively. At unstimulated baseline (Fig. 6b), older cDC1 had significantly impaired phagocytosis (*p* = 0.0003) (Fig. 6b). After stimulation with LPS/IFN-γ and RIG-I, cDC1 from older donors still had significantly decreased phagocytic activity when compared with adult counterparts (LPS/IFN-γ, *p* = 0.0116; RIG-I, *p* = 0.0022). Importantly, in response to the RIG-I agonist, phagocytosis in cDC2 from older donors was decreased (*p* = 0.0002) (Fig. 6c). Phagocytosis by monocyte subsets is depicted in Fig. 6d and e. We observed significant decreases in phagocytosis in CD14^dim^CD16^+^ monocytes of older donors in response to the G10 STING agonist (*p* < 0.0001) (Fig. 6d). We also observed a significant increase in phagocytosis in CD14^+^CD16^−^ classical monocytes in older donors compared with adult counterparts (*p* = 0.0495) after STING stimulation (Fig. 6e). These data suggest an induced defect in the function of DC and monocyte subsets of older individuals which impacts vaccine efficacy and infection.

## Discussion

It is a widely accepted fact that aging is associated with increased morbidity and mortality to many viral infections, the most notable today being SARS-CoV-2 or COVID [38]. Multiple factors contribute to this increase in susceptibility including impaired vaccine responsiveness and creation of immunological memory [3, 39]. While the adaptive immune response to aging has been extensively studied, the more important branch of immunity, innate immunity, has remained incompletely and poorly understood [3, 4, 40]. We have previously reported that IFN production and gene expression in response to PRR agonist stimulation are distinctly impaired in peripheral blood mononuclear cells (PBMCs) and monocyte subsets from older, healthy human donors [6, 7]. To develop successful therapeutic strategies for this vulnerable population, it is essential to further understand the underlying nature of this defect. Using primary PBMCs from older and adult healthy donors, we demonstrated that cells from older participants exhibit a defect in type I IFNs and IL-1β and in cytokines important for T cell differentiation such as IL-6 and IFN-γ (Fig. 1). We have also identified three main defects in innate antiviral signaling that align with this decrease in IFN production, each denoting a different antiviral pathway. Decreased phosphorylation of IRF7 and TBK-1 in CD14^dim^CD16^+^ monocytes, cDC1, and cDC2 from older donors impairs the production of IFN downstream of TLR7/8 and RIG-I pathways in primary antiviral responses and the ability of these cells to respond quickly to viral infection. TBK-1 not only induces innate antiviral type I IFNs but also has much broader functions including support for efficient antibody responses and autophagy. Recent studies have shown that TBK-1 associates with inducible T cell co-stimulator (ICOS) and is involved in the differentiation of germinal center (GC) T^FH^ cells and the development of B cell responses [41]. Consequences of decreased TBK-1 activity due to aging may curtail humoral immunity through this ICOS-driven GC pathway. Of note, we and others have not seen any impairment in TNF-α production following stimulation with TLR or RLR agonist. This led us to postulate that NF-κB and mitochondrial antiviral-signaling protein (MAVS) signaling is still intact in the elderly This led us to postulate that NFkB and mitochondrial antiviral-signaling protein (MAVS) signaling is still intact in older adults. [32, 42]. We also show that this weakened response leads to dampened activation of STING in the cGAS-STING pathway. STING has been shown to regulate an array of innate immunity pathways including autophagy and other cell death pathways like apoptosis. STING-dependent autophagy responses are important for clearing DNA from the cytosol and for activating protective responses against viruses such as West Nile and dengue viruses [43]. Lowered STING activation with aging could be contributing to less effective viral clearance mechanisms [44]. These data corroborate that older population has a delayed early phase and non-productive late phase of the response to pathogens, resulting in increased disease severity and poor vaccination responses.

We show that the impairment of IFN production affects monocyte and DC subset phagocytic ability in response to agonist. We believe that the lack of IFN production leads to dampening of the IFN feed-forward loop prompting type II IFN, IFN-γ, impairment (Fig. 1b). IFN-γ is a regulator of the critical antiviral immune response and affects the phagocytic function of monocytes, ILCs, and DCs and mediates early attrition of existing memory CD8^+^ T cells in response to viral infections in mice and humans [45–47]. This function is critical not only to achieving a rapid and efficient clearance of viral pathogens, but also to initiating antigen presentation and IL-12 production [11]. In this study, we see a reduction in the phagocytic ability of CD14^dim^CD16^+^ monocytes, cDC1, and cDC2s (Fig. 6). CD14^dim^CD16^+^ monocytes respond to viral infection in the endothelium and are common responders to respiratory viral infections. A deficit in type I IFN secretion and phagocytosis in this cell type partially explain older adults’ susceptibility to infection including respiratory pathogens like SARS-CoV-2 and influenza. We also show that cDC1s have defective signaling in TLR7/8, RIG-I, and STING pathways at early and late time points (Figs. 3, 4, and 5) which corresponds with less efficient phagocytosis. These data have direct implications for adaptive immunity in older adults in response to viral pathogens that do not infect DCs directly, suggesting potential impairment of cross-presentation and activation of CD8^+^ T cells or polarization of CD4^+^ T cells through the secretion of IL-12 [11, 34]. Additionally, we saw that antiviral pathway activation was decreased in cDC2s from older adults. Several reports have shown that cDC2s are the dominant Tfh-priming DC subset, due to their ability to traffic to GCs where they occupy the T-B border regions in secondary lymphoid organs [48–52]. Defects in this function are likely to result in deficits in antigen presentation by cDC2s to T cells and, subsequently, an inability to respond productively to a viral infection. More research is needed to further elucidate how aging shapes the cytokine milieu in the GC for Tfh priming in the context of human DC and monocyte subsets. Paradoxically, phagocytosis was increased in classical CD14^+^CD16^−^ monocytes in older individuals. Previous studies in our lab have shown that CD14^+^CD16^−^ monocytes from older individuals have significantly higher expression of C-X3-C motif chemokine receptor 1 (CX3CR1) under non-stimulated conditions and, therefore, could be trafficking with greater efficiency to tissues and contributing to degenerative effects in the heart and other organs [7]. Taken together, these results demonstrate that the ability of older adults to induce IFNs is compromised in multiple antiviral pathways, which, in turn, affects the efficiency of phagocytosis, to the detriment of antiviral clearance mechanisms. This introduces potential targets in these pathways toward the goal of restoring efficient antiviral immunity or overcoming these deficits via a prospective vaccination strategy.

Unlike cDCs, pDCs showed no significant difference in the phosphorylation of TLR7/8 and TBK-1 or in the upregulation of STING in response to pathway-specific agonists (Supplemental Figs. 5 and 6). Given that pDCs produce large amounts of type I IFN, the initial IFN defect in older individuals might be secondary to downstream immunological functions such as phagocytosis and, ultimately, antigen presentation. Decreased phagocytic capacity would result in decreased antigen presentation to CD8^+^ and CD4^+^ cells, including Tfh cells in the GC. This diminished function would not only explain the age-related discrepancy in antiviral response but could prove to be a valuable target in therapeutics aiming to ameliorate the dampened antigen presentation in older individuals. Future studies should aim to shed light on this potential deficit. However, it is possible we have not seen changes in this subset due to their lower frequencies in PBMCs.

Learning more about how aging shapes cellular sensing by TLR and RLR pathways will aid in developing therapeutic strategies to normalize responses to vaccines by adjuvantation as well as infection. Two of the FDA-approved COVID-19 vaccines utilize lipid nanoparticle (LNP) technology to induce effective immune responses; however, the mechanism of action for this vaccine platform is not well characterized. Existing LNP research has shown that these nanoparticles alone, without mRNA, elicit sensing through MyD88 and MAVS. More study into this technology should aim to elucidate pathways triggered in monocytes and DCs [53]. The delineation of specific monocyte and DC subsets is a significant advancement in our understanding of aging innate immunology. Due to the diverse range of roles and functions played by these subsets, broadly labeling them as one group would not do justice to their importance.

**Fig. 7:**
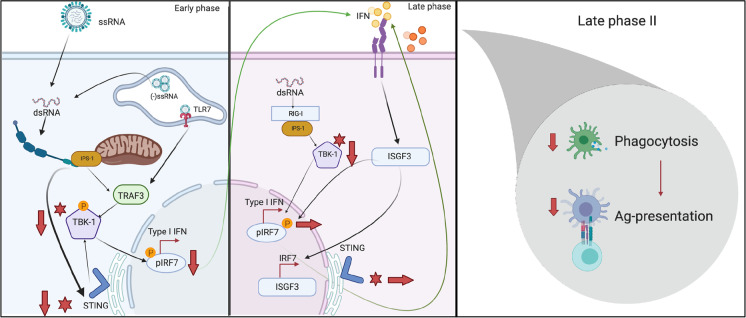
Model of age-induced impairment of IFN signaling in monocyte and DC subsets. During the initial IFN response (within minutes) due to ssRNA, dsRNA, or cytosolic DNA stimulation or infection, the phosphorylation of IRF7 and TBK-1 or the activation of STING is impaired in older donors when compared to young counterparts. Once TBK-1 has been phosphorylated, it goes on either to activate STING or to stimulate IFN, or initiates the dimerization and phosphorylation of IRF7 to be translocated to the nucleus to stimulate the IFN response. Aging has been associated with decreased type I IFN secretion. We hypothesize that this impairment is due to the decreased phosphorylation and activation of key signaling molecules that lead to an impairment in the late phase which occurs at 24 h post stimulation. During late phase II, impairment of TLR7/8, RIG-I, and cGAS-STING signaling continued, compromising the immunological functional roles such as phagocytosis

In conclusion, our results demonstrate three key defects in innate signaling that together play a role in the impairment of IFN-mediated antiviral response and subsequent antiviral function in monocyte and DC subsets in older individuals (Fig. 7). First, we show that the decreased phosphorylation of TBK-1, a kinase that phosphorylates IRF7, IRF3, and NF-κB, results in reduced phosphorylation of the transcription factor, IRF7, in the TLR7/8 and RIG-I pathways in CD14^dim^CD16^+^ monocytes, cDC1, and cDC2. We further show that this results in alteration of the cGAS-STING and RIG-I pathways within these cell subsets. Finally, we show pathway-specific agonist compounds upon immune function, resulting in decreased phagocytosis by CD14^dim^CD16^+^ monocytes, cDC1, and cCD2 from older adults. By illuminating new avenues of investigation, therapeutic strategies targeting IRF7 or TBK-1 can be developed to boost vaccine responses to viral infections such as influenza and SARS-CoV-2 to alleviate substandard vaccine outcomes in the older population.

## Supplementary Information

Below is the link to the electronic supplementary material.

Supplementary file1 (PDF 2540 KB) Supplemental Table 1 Patient recruitment and enrollment. Healthy. community dwelling subjects were enrolled from Martin Health System. Subjects were excluded who reported comorbid conditions including cancer, immunocompromising disorders, and steroid use [7]. Supplemental Fig. 1 Flow cytometry gating strategy. **a**. DC subsets are as follows: “pDC” HLA-DR+, CD1c+, CD11c+, CD303+CD141-, “cDC1” HLA-DR+, CD1c+, CD11c+, CD303-CD141+, “cDC2” HLA-DR+, CD1c-, CD11c-, CD303-CD141-. **b**. Monocyte subsets are as follows: “classical” HLA-DR+. CD14+CD16-, “intermediate” HLA-DR+, CD14+CD16+CD16-, “non-classical” HLA-DR+ CD14dimCD16+CD16-. Supplemental Fig. 2 TLR7 expression on surface of DC and monocyte subsets. Human PBMCs, monocyte subsets, and DC subsets from the blood of younger (age, 24-36 years; n = 11) and older (age, 67-83 years; n = 11), healthy donors were ex-vivo stained for the baseline expression of TLR7. Monocyte subsets (**a**) and DC subsets (**b**) were permeabilized, fixed, and stained for TLR7. After gating on DC and monocyte subsets, the median intensity (MFI) was measured using flow cytometry. Data are means. *P<0.05, **P < 0.01, ***P < 0.001 non-parametric Mann Whitney test. Supplemental Fig. 3 Some cytokines were not significantly correlated independent of age. (**a**-**d**) Supernatant was collected from PBMCs of all donors 24 hours after stimulation and analyzed by Luminex assay to measure IFN-α, IFN-γ, IL-1β, TNF-α, or IL-6 secretion. All concentrations were measured using the human Procarta Plex 5-Plex from Invitrogen. Each point represents one donor. Pearson’s correlation test was used for all correlation graphs. Data are representative of one experiment with n=9/group. *P<0.05, **P < 0.01, ***P < 0.001, ****P < 0.0001. Supplemental Fig. 4 pIRF7 and pTBK-1 is not significantly different between age groups at baseline. Human PBMCs, monocyte subsets, and DC subsets from the blood of younger (age, 24-36 years; n = 11) and older (age, 67-83 years; n = 11), healthy donors were assessed for their phosphorylation status of (**a**) IRF7 and (**b**) TBK-1 at unstimulated, baseline levels. Cells were incubated at 37 °C for 24 hours, permeabilized, fixed, and stained for TLR7. After gating on monocyte and DC subsets, the geometric mean intensity (MFI) was measured using flow cytometry. Data are means *P<0.05, **P < 0.01, ***P < 0.001 unpaired, non-parametric Mann Whitney test. Supplemental Fig. 5 Phosphorylation of IRF7 or TBK-1 was not significantly different in pDCs. Human PBMCs, monocyte subsets, and DC subsets from the blood of younger (age, 24-36 years; n = 11) and older (age, 67-83 years; n = 11), healthy donors were either stimulated with LPS/IFNγ or CLO97, a TLR7/8 specific agonist, G10, a cGAS-STING specific agonist, or transfected with a RIG-I-specific 3p-hpRNA ligand. PBMCs were stimulated or left unstimulated for 15 or 45 min or 24 hours. Cells were permeabilized, fixed, and stained for (**a**) pIRF7 (**b**) pTBK-1 or (**c**) STING. After gating on monocyte and DC subsets, the geometric mean intensity (MFI) was measured using Phosflow cytometry. Data are means *P<0.05, **P < 0.01, ***P < 0.001 unpaired, non-parametric Mann Whitney test. Supplemental Fig. 6 Phagocytosis was not significantly different in pDCs or intermediate CD14+ CD16+ monocytes. **a-b**. Phagocytosis measured using fluorescent beads and multiparametric flow cytometry. PBMCs were incubated overnight with stimulus and with beads for a further 3 hours. MFI of fluorescent beads of indicated subsets from young (n=12) and old (n=12) patients is indicated. Data is from two independent experiments and are shown (mean ± sem.). P values were determined using non-parametric Mann Whitney test.

## Data Availability

All the data supporting the findings of this study are available within the article and its Supplementary information files or from the corresponding author upon reasonable request.
