# Supplementary material for: Aging alters antiviral signaling pathways resulting in functional impairment in innate immunity in response to pattern recognition receptor agonists
Source: GeroScience. 2022 Jul 18;44(5):2555–72. doi: 10.1007/s11357-022-00612-5 (PMC9289086; doi:10.1007/s11357-022-00612-5)
Supplement: Supplementary file 1 — Supplementary file1 (PDF 2540 KB) Supplemental Table 1 Patient recruitment and enrollment. Healthy. community dwelling subjects were enrolled from Martin Health System. Subjects were excluded who reported comorbid conditions including cancer, immunocompromising disorders, and steroid use [7]. Supplemental Fig. 1 Flow cytometry gating strategy. a. DC subsets are as follows: “pDC” HLA-DR+, CD1c+, CD11c+, CD303+CD141-, “cDC1” HLA-DR+, CD1c+, CD11c+, CD303-CD141+, “cDC2” HLA-DR+, CD1c-, CD11c-, CD303-CD141-. b. Monocyte subsets are as follows: “classical” HLA-DR+. CD14+CD16-, “intermediate” HLA-DR+, CD14+CD16+CD16-, “non-classical” HLA-DR+ CD14dimCD16+CD16-. Supplemental Fig. 2 TLR7 expression on surface of DC and monocyte subsets. Human PBMCs, monocyte subsets, and DC subsets from the blood of younger (age, 24-36 years; n = 11) and older (age, 67-83 years; n = 11), healthy donors were ex-vivo stained for the baseline expression of TLR7. Monocyte subsets (a) and DC subsets (b) were permeabilized, fixed, and stained for TLR7. After gating on DC and monocyte subsets, the median intensity (MFI) was measured using flow cytometry. Data are means. *P<0.05, **P < 0.01, ***P < 0.001 non-parametric Mann Whitney test. Supplemental Fig. 3 Some cytokines were not significantly correlated independent of age. (a-d) Supernatant was collected from PBMCs of all donors 24 hours after stimulation and analyzed by Luminex assay to measure IFN-α, IFN-γ, IL-1β, TNF-α, or IL-6 secretion. All concentrations were measured using the human Procarta Plex 5-Plex from Invitrogen. Each point represents one donor. Pearson’s correlation test was used for all correlation graphs. Data are representative of one experiment with n=9/group. *P<0.05, **P < 0.01, ***P < 0.001, ****P < 0.0001. Supplemental Fig. 4 pIRF7 and pTBK-1 is not significantly different between age groups at baseline. Human PBMCs, monocyte subsets, and DC subsets from the blood of younger (age, 24-36 years; n = 11) and older (age, 67-83 y [file 11357_2022_612_MOESM1_ESM.pdf]

Supplemental Table 1: Patient recruitment and enrollment.

	Adults (n=11)	Older subjects (n=11)
Average age (range), y	30 (24-36)	73 (67-83)
Gender, male/female (female %)	4/7 (64)	5/6 (55)
Race, n		
White (non-Hispanic)	9 (82%)	11 (100%)
White (Hispanic)	2 (18%)	
Comorbidities, n	1 (9%)	9 (82%)
None	10 (91%)	2 (18%)
Arthritis	1 (9%)	5 (45%)
Hypertension		5 (45%)
Stroke		1 (9%)
Heart disease		1 (9%)
Medications		
Prescription	1 (9%)	6 (55%)
Over-the-counter	5 (45%)	8 (73%)

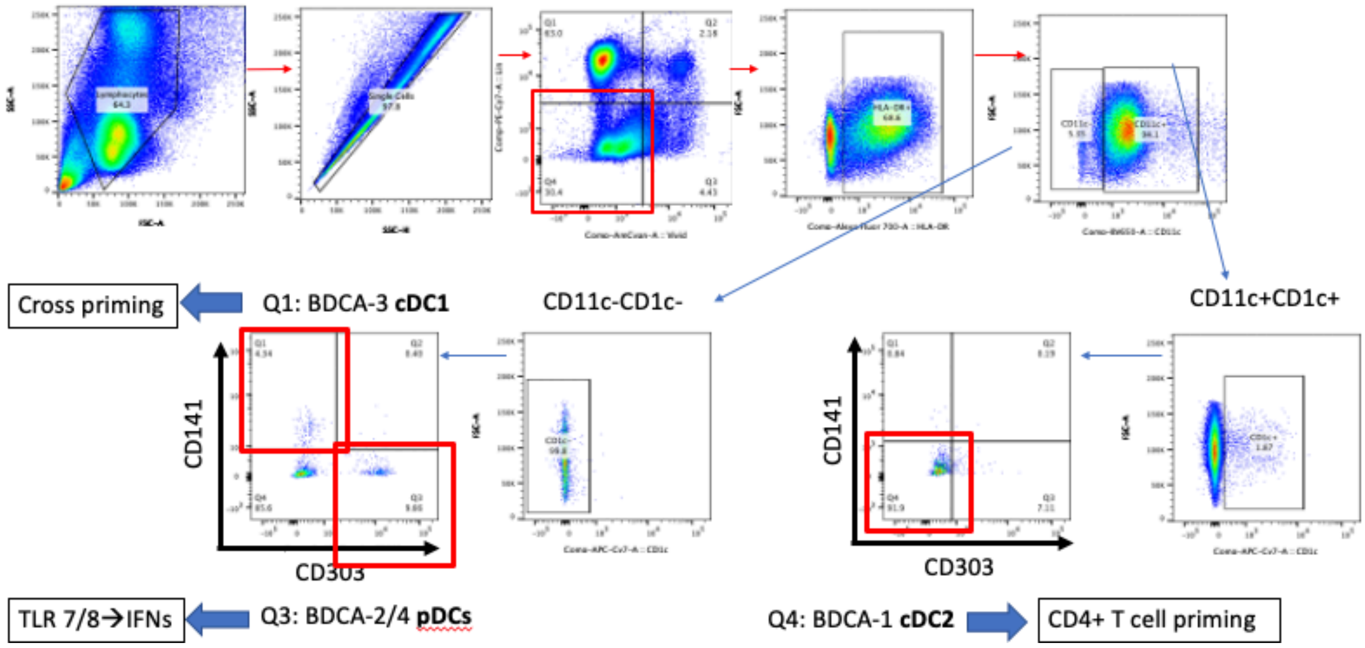

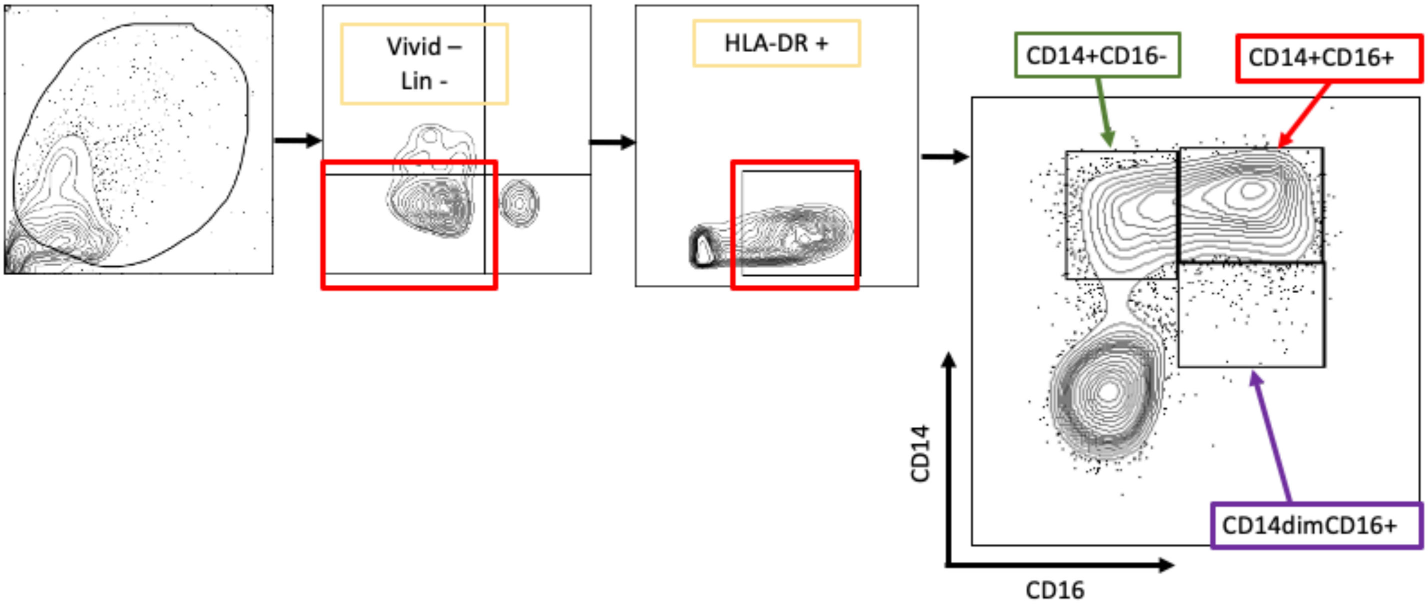

Supplemental fig 1 :Flow cytometry gating strategy

Supplemental table 2: Flow cytometry antibody list

Antigen	Clone	Fluorophore
CD3	HIT3α	PE-Cy7
CD56	5.1H11	PE-Cy7
CD19	HIB19	PE-Cy7
CD20	2H7	PE-Cy7
CD11c	BU15	BV605
CD14	M5E2	PE-Tx Red
HLA-DR	L243	AF700
CD16	3G8	BV650
CD1c	L161	APC-Cy7
CD303	201A	PerCP5.5
pIRF7	K47-671	APC
pTBK-1	J133-587	PE
STING	T3-680	BV421
CD141	AD5-14H12	FITC

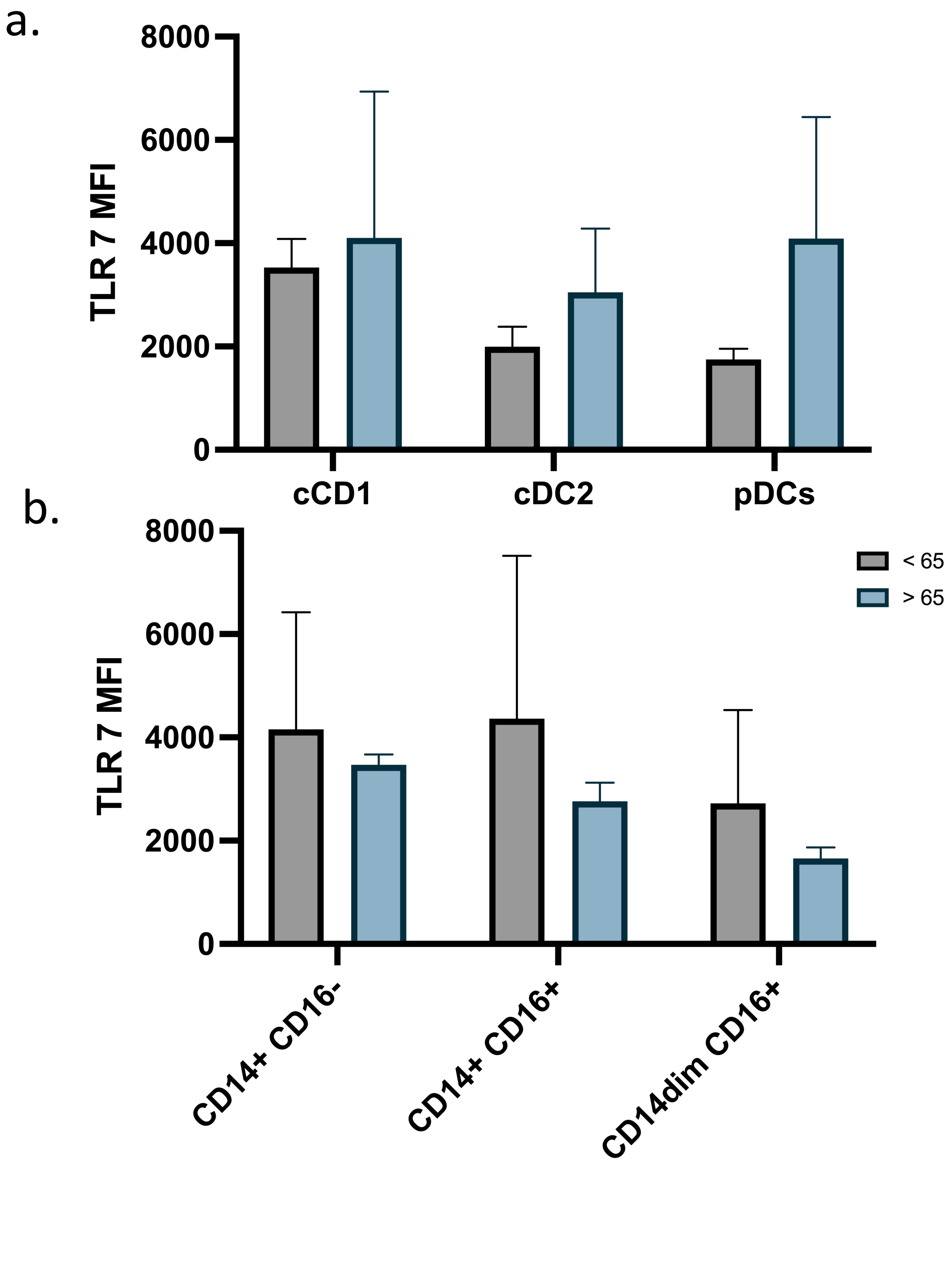

Supplemental fig 2 :TLR7 expression on surface of DC and monocyte subsets

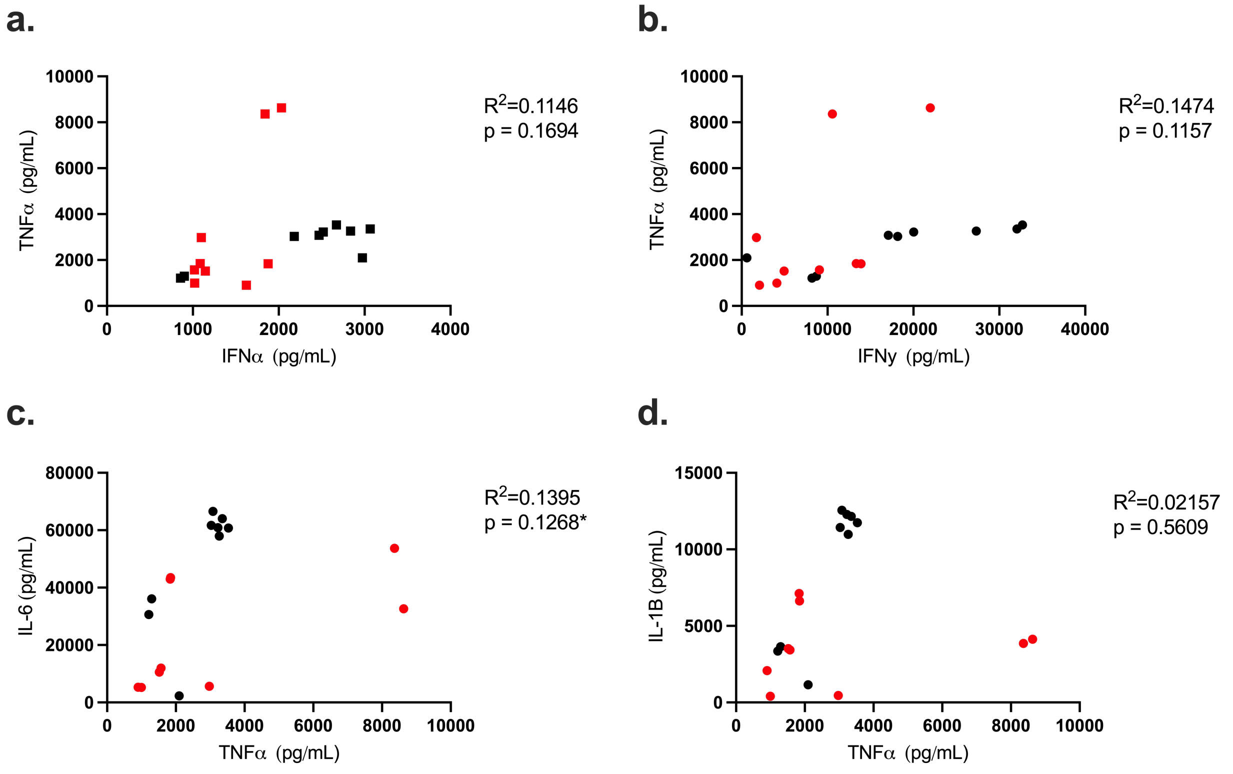

Supplemental fig 3: Some cytokines were not significantly correlated independent of age

a.

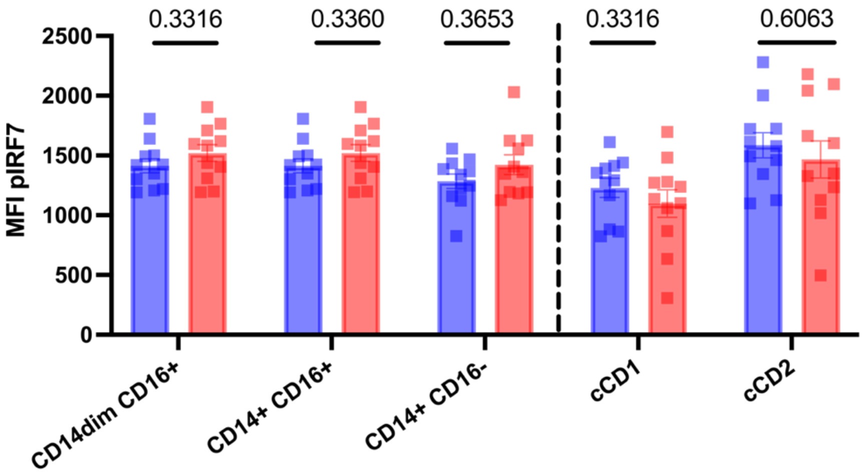

b.

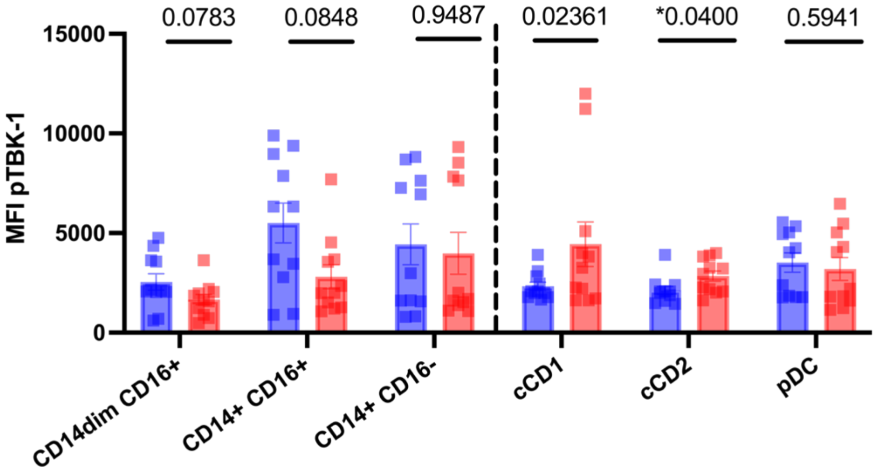

Supplemental fig 4: pIRF7 and pTBK-1 is not significantly different between age groups at baseline

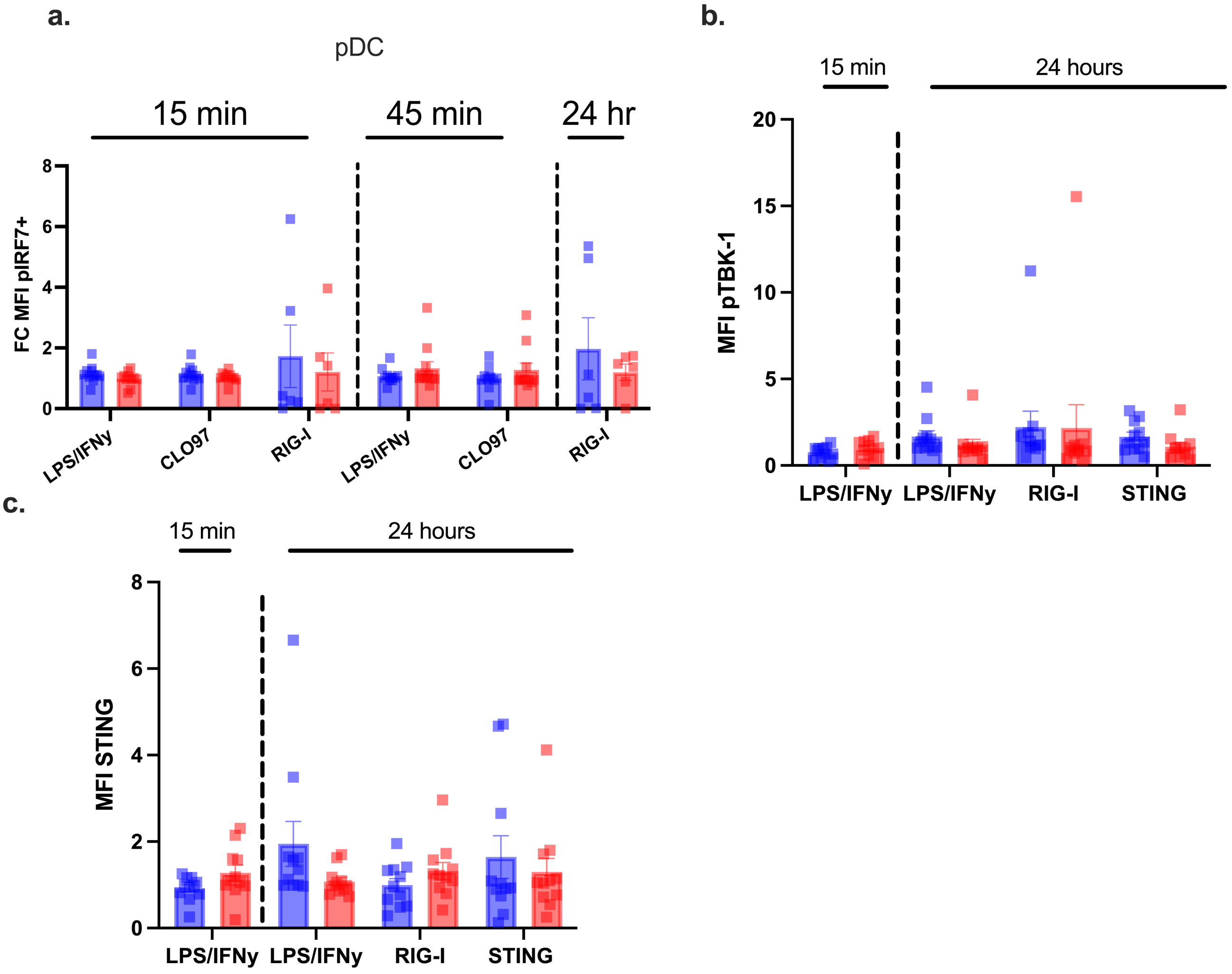

Supplemental fig 5: **Phosphorylation of IRF7 or TBK-1 was not significantly different in classical monocytes or pDCs.**

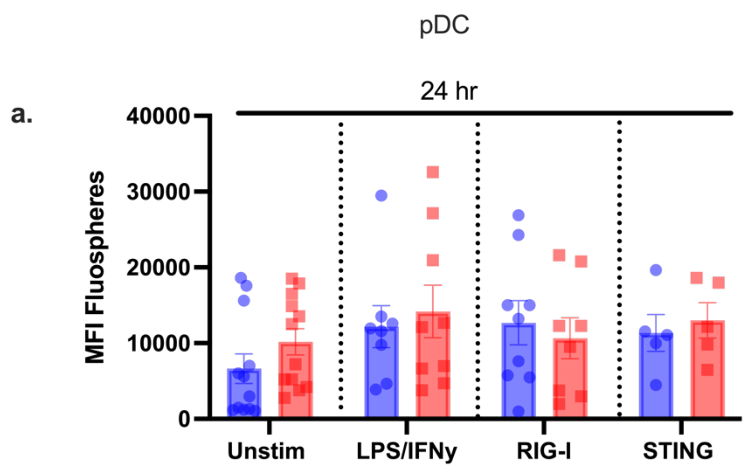

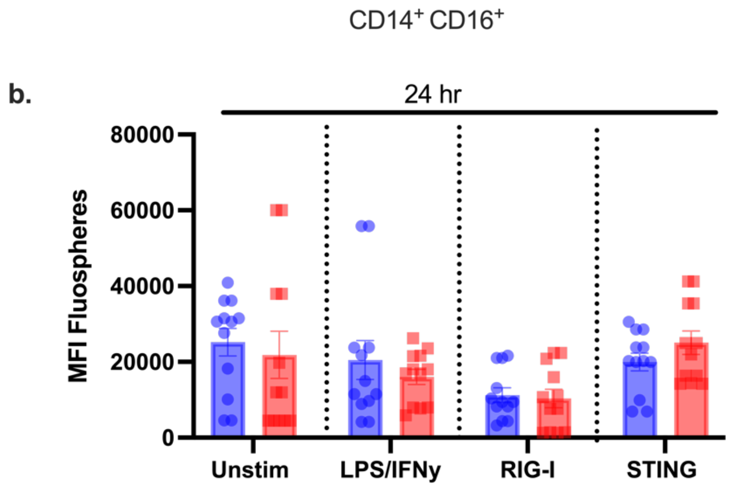

Supplemental fig 6 : Phagocytosis was not significantly different in pDCs or intermediate CD14+ CD16+ monocytes
